# The relationship between a child’s postural stability and manual dexterity

**DOI:** 10.1007/s00221-014-3947-4

**Published:** 2014-05-14

**Authors:** Ian Flatters, Faisal Mushtaq, Liam J. B. Hill, Raymond J. Holt, Richard M. Wilkie, Mark Mon-Williams

**Affiliations:** 1 Institute of Psychological Sciences, University of Leeds, Leeds, West Yorkshire UK; 2 School of Mechanical Engineering, University of Leeds, Leeds, West Yorkshire UK

**Keywords:** Fine and gross motor control, Posture, Manual dexterity, Visuomotor, Kinematic, Motor development

## Abstract

The neural systems responsible for postural control are separate from the neural substrates that underpin control of the hand. Nonetheless, postural control and eye-hand coordination are linked functionally. For example, a stable platform is required for precise manual control tasks (e.g. handwriting) and thus such skills often cannot develop until the child is able to sit or stand upright. This raises the question of the strength of the empirical relationship between measures of postural stability and manual motor control. We recorded objective computerised measures of postural stability in stance and manual control in sitting in a sample of school children (*n* = 278) aged 3–11 years in order to explore the extent to which measures of manual skill could be predicted by measures of postural stability. A strong correlation was found across the whole sample between separate measures of postural stability and manual control taken on different days. Following correction for age, a significant but modest correlation was found. Regression analysis with age correction revealed that postural stability accounted for between 1 and 10 % of the variance in manual performance, dependent on the specific manual task. These data reflect an interdependent functional relationship between manual control and postural stability development. Nevertheless, the relatively small proportion of the explained variance is consistent with the anatomically distinct neural architecture that exists for ‘gross’ and ‘fine’ motor control. These data justify the approach of motor batteries that provide separate assessments of postural stability and manual dexterity and have implications for therapeutic intervention in developmental disorders.

## Introduction

The motor control literature often differentiates between ‘gross’ and ‘fine’ motor control. The term ‘gross motor’ control is used generally to describe activities involving locomotion and movement of the torso (e.g. walking, maintaining postural stability). Whereas ‘fine motor’ control is associated with tasks that typically involve some form of manual manipulation (Malina et al. 2004). Many standardised assessments of childhood motor performance reflect this division in their design and subscales. For example, the Movement ABC-2 comprises of three sets of tasks, each set tailored to assess one of the following subcomponents of motor control: ‘manual dexterity’, ‘aiming and catching’ and ‘balance’ (Henderson et al. 2007). The justification for compartmentalising motor control performance into these subcategories is not clear. Henderson and Barnett (1998) state that it follows an: ‘agreed taxonomy’ but this agreement is based only on subjective ‘common sense and clinical experience’. Until recently, there has been little empirical evidence to justify assessing motor skills along such lines (Schulz et al. 2011).

One could argue that categorising any motor action as either ‘fine’ or ‘gross’ is overly simplistic, given that many motor tasks require fine *and* gross activity in conjunction. From infancy, skilled postural control is a prerequisite for the acquisition of optimal reaching and grasping behaviours (De Graaf-Peters et al. 2007; Lobo and Galloway 2008). Postural stability moderates the rate at which infants learn successful grasping (Cunha et al. 2013) and reaching is comparatively impaired in infants who have not yet developed the compensatory head and trunk movements required to counterbalance their arm movements during such behaviour (von Hofsten 1982; Amiel-Tison and Grenier 1983; De Graaf-Peters et al. 2007). Even in adulthood, postural stability is found to vary as a function of the level of precision required during a concurrent manual control task (Haddad et al. 2010).

The interaction between postural control and arm movements raises fundamental questions about the relationship between an individual child’s ability with regard to postural control and their fine motor skills (in particular between postural stability and manual dexterity). There are three broad positions with respect to the relationship between these skills that are mutually exclusive and can be formally outlined as follows:

- Position I: postural control and fine motor skills are completely independent developing processes requiring absolute taxonomic separation when examining performance (Henderson and Barnett 1998).
- Position II: postural control and fine motor skills are highly correlated attributes that reflect an underlying ability (a postulated ‘motor ability’ construct).
- Position III: postural control and fine motor skills are separate processes that, nonetheless, affect each other’s development through their co-dependent functional combination across various tasks (Haddad et al. 2013).

It is clear that the neural circuits that support posture and the circuits that underpin manual control are functionally modular. Functional modularity does not imply anatomical modularity as the different systems share at least some of the same neural substrates (e.g. motor cortex), make use of common computational resources and are distributed across multiple levels of the central nervous system. Moreover, a number of everyday tasks require coordinated patterns of activity (e.g. postural responses that respond to changes in the centre of gravity wrought by arm movements, see Flatters et al. 2014c). It is, therefore, impossible to use neural anatomy to determine the extent to which manual dexterity is predicted by postural control ability in childhood.

Logically, it is possible that the neural systems underpinning posture and manual control develop independently (Position I). Children who experience difficulties in motor development often have a deficit in fine, but not gross motor skills (Visser 2003; Zwicker et al. 2012) or vice versa children with spina bifida are unable to stand but when seated are able to perform manual control tasks, implying that distinct and independent neural substrates may be responsible for each skill’s development. This interpretation: (i) agrees with research that shows gross, but not fine motor skills in infancy are a significant predictor of cognitive performance at school age (Piek et al. 2008) and (ii) reports of both boys and girls showing isolated advantages on specific motor tasks (Thomas and French 1985; Junaid and Fellowes 2006; Smith et al. 2012). The independence of gross and fine motor skill development is further supported by evidence that their trajectories (from infancy to preschool) are best described by different mathematical models (Darrah et al. 2009).

Motor skill development in general follows a nonlinear and discontinuous trajectory (Riach and Starkes 1994; Kuhtz-Buschbeck et al. 1998), punctuated by the accomplishment of increasingly complex hierarchical ‘motor milestones’ (WHO 2006). These milestones present as emergent behaviours generated by a number of interconnected processes. For example, over-arm throwing is initially a predominantly upper limb action that matures over time to incorporate more gross-locomotor aspects (e.g. a step phase and rotation of hips, torso and shoulder prior to release; Malina et al. 2004). Such an observation implies that postural and fine motor control may be independent dynamical processes, which in the course of development often create more complex ‘higher level’ coordinated motor actions.

It is therefore also logically possible that the creation of coordinated motor actions has a profound effect on the individual development of the different systems. A primary role of the human postural system appears to be to provide the stability necessary to obtain reliable visual information, which is vital for guiding skilful manual interactions with the world (Thelen and Spencer 1998; Fallang et al. 2005; Haddad et al. 2013). Thus, an integrated role for posture in the function and development of manual dexterity makes sense from a mechanical perspective. This is illustrated by considering an imminent volitional movement to reach for an object. The postural system generates preemptive inertia from the displacement of the centre of mass, opposed in direction and magnitude to the inertia generated by the hand movement. This anticipatory postural adjustment (APA) results in a cancellation of the force generated by the hand movement and minimises the centre of mass (COM) displacement (Massion 1992). The integration of postural and fine motor control through APAs becomes more proficient over childhood and allows for the development of increasingly complex and skilled manual control behaviours (Van Der Fits et al. 1999). Such a close functional relationship could ultimately produce highly correlated skill levels in posture control and manual dexterity to the point where it becomes meaningful to discuss a single ‘motor skill’ construct (Position II). Alternatively, the two processes might impact on the development of the other but to a lesser extent (Position III)—so that a developmental relationship exists between the processes, whilst they clearly maintain their own individual trajectories.

Empirical data are required to test between Position I, II and III but studies directly testing the strength of association between children’s gross and fine motor control skills are scarce. Moreover, those that do exist report very mixed findings in relatively small sample sizes. In infants, Loria (1980) found no correlation between reaching and prehensile skills in a sample of twelve 30-week-old children using objective observational rating methods. Case-Smith et al. (1989) measured a sample of 60 children aged between 2 and 6 months old on the posture and fine motor assessment of infants (PFMAI) scale and found scores for posture only accounted for 12 % of the variance in fine motor control scores. In contrast, Wang et al. (2011) found that in a sample of 105 six-to-twelve-month-old preterm infants postural control, assessed using the Alberta Infant Motor Scale, was a significant predictor uniquely explaining 25 % of the variance in fine motor control, assessed using subtests from the Peabody Developmental Motor Scales. Beyond infancy, Rosenblum and Josman (2003) examined fine motor performance using a peg-board manual dexterity task and a set of balance tasks from the Bruininks-Oseretsky Test of Motor Proficiency (BOTMP), in 47 five-year-old children. They found small-to-moderately sized correlations between some of the fine motor and postural stability outcomes (ranging from *r* = −0.31 to −47), but these results were affected by ceiling effects on some measures and statistical analysis did not adjust for multiple comparisons. Two studies have looked for relationships between proximal muscle activation (underpinning posture) using electromyography and performance levels on pencil-paper handwriting and drawing tasks: Wilson and Trombly (1984) showed no relationship between magnitude of (gross motor) muscle activation and quality of performance on two standardised assessments of fine motor control in a sample of sixteen 6–8 year olds. In contrast, Naider-Steinhart and Katz-Leurer (2007) found that decreased variability in both proximal (trapezius) and distal (thumb) muscle activity was associated with faster handwriting speeds in a sample of thirty-five 8–10 year olds.

The existing literature has utilised relatively unsophisticated assessments of gross and fine motor control that are too time consuming to be employed in large population-based samples. Furthermore, these tools tend to produce noisy estimates of ability because they rely either on observational judgements, simplistic scoring criteria (e.g. ‘pass/fail’ judgements) and/or require participants to produce unfamiliar behaviours that lack ecological validity (e.g. standing on one leg for an extended period of time). These issues are particularly problematic if one wants to conduct research in large samples and detect subtle variations in task performance (Culmer et al. 2009). To address these issues, we used a postural measurement rig capable of providing accurate and reliable quantitative measures of postural behaviour in children across the primary school age range (Flatters et al. 2014a). In conjunction with this set-up, we also used a computerised battery of manual fine motor control tests: the Clinical Kinematic Assessment Tool (C-KAT) to provide detailed kinematic investigations of end point control across a range of subtests including tracking, sequential aiming and tracing tasks (Culmer et al. 2009). This software platform has been used experimentally as a tool for investigating motor learning and manual control in a number of previous studies (Johnson et al. 2010; Gonzalez et al. 2011; Raw et al. 2012a, b). We have shown previously that these tools capture large changes in postural control (Flatters et al. 2014a; Flatters et al. in press) and manual control (Flatters et al. 2014b) as a function of age. We reasoned that testing a large number of children on these objective measures of motor control would allow us to detect associations between postural stability and manual motor performance across the age groups but also explore the relationship after we controlled for age.

## Methods

### Participants

All children registered at two mainstream schools (total of 517 students) in the West Yorkshire region (United Kingdom) were invited to participate in the research. On the first testing session, we collected data from 495 children (235 male, 260 female, age range 3 years 2 months to 12 years 2 months, mean age = 7 years 2 months) on the manual control measure (22 children were either absent from school or opted out of testing). On the second session, we randomly selected classes across both schools within each year group and measured postural control in students from these classes. The final sample, with data recorded on postural stability in stance *and* manual control in sitting, comprised of 278 children (134 male, 144 female, age range 3 years 2 months to 11 years 10 months, mean age = 7 years 8 months). There were no children with severe disability within the schools, but it is probable that there were a number of children with neurodevelopmental problems (e.g. autism). We randomly sampled classes across both schools and did not exclude any child from these classes from participating, so our data are likely to be reasonably representative of a typical UK school population. Ethical approval for this study was obtained from the University of Leeds Ethics and Research committee.

### Manual control measures

Participants completed a battery of fine motor tests called the Clinical Kinematic Assessment Tool (C-KAT). The C-KAT software, which is used to present the battery (Culmer et al. 2009), was created using a software development environment: LabVIEW (version 8.2.1, National Instruments TM) deployed on a Toshiba digitizing tablet portable computer (Toshiba Portégé, 14″ screen: 260 × 163 mm, 1,280 × 800 pixels, 32 bit colour, 60 Hz refresh time). The tablet’s screen provides a horizontal surface (in landscape orientation) similar to writing with a pen and paper using a stylus as an input device. Participants were seated at a desk with the tablet placed in front of them, 10 cm in from the table edge.

**Fig. 1:**
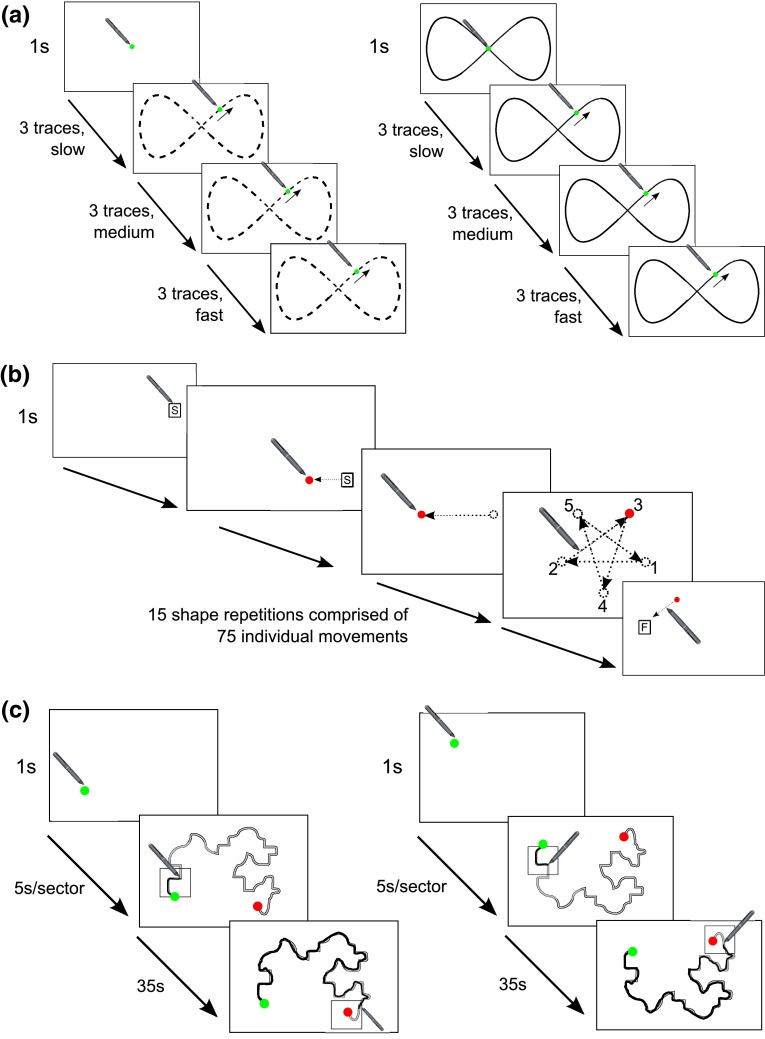
Illustration of the three C-KAT battery tasks: **a** *left* is a schematic of first tracking trial (i.e. without ‘Guideline’), annotated with a *dotted line* to indicate the trajectory of the moving dot. *Right* is a schematic of the second tracking trial, which included the additional guideline; **b** schematic of the aiming subtest, annotated with *dotted arrows* implying the movements participants would make with their stylus to move off the start position, between target locations and to reach the finish position, the 4th panel’s annotations indicating the locations and order in which targets sequentially appeared; **c** *left* is a schematic depicting tracing path A and *right* is a schematic depicting tracing path B. The *black shaky lines* are an example of the ‘ink trails’ a participant would produce with their stylus in the course of tracing

The C-KAT battery comprised of three visuo-spatial subtests completed in the following fixed order: *tracking:* participants placed their stylus, for 2 s, on a stationary green dot (10 mm in diameter) presented on the tablet screen, triggering it to begin moving around the screen by doing so. The dot’s movements followed a horizontal ‘figure-of-8’ spatial pattern (see Fig. 1a) continuously for 84 s (nine revolutions) and as it did so participants were asked to try and keep their stylus as close to the centre of it as possible. The motion was described by two oscillating sinusoidal waveforms in the axes of the screen.

These had a 2:1 ratio with respect to their frequencies and amplitudes, resulting in a ‘figure-8’ of 55 mm in height and 110 mm in width. This trial was repeated twice, first with no guide and then with a background ‘guideline’ provided to give the participant additional information on the spatial path the dot followed (see Fig. 1a). In each trial, the target dot’s speed immediately increased after a trio of revolutions. Starting ‘slow’ and increasing to ‘medium’ the ‘fast’ speeds (velocities of 42, 84 and 168 mm/s, respectively).

*Aiming*: in this subtest, participants were required to move from one target dot to another without lifting the stylus from the screen. The trial began when participants placed the stylus on the ‘start’ button for 2 s; this prompted the first target dot to appear. When the first target dot was reached, it disappeared and another appeared in a different location on the screen. Participants were instructed to move successively from one target dot to the next until they had made 75 aiming movements, after which a ‘finish’ block appeared. The trial terminated once they moved their stylus to it. Within these 75 movements, the participants moved successively to five fixed targeted locations (numbered in Fig. 1b) before being re-cued to the first location and repeating this sequence (i.e. participants resultant movements approximated drawing the star shape outlined in the fourth panel of Fig. 1b fifteen times in a row). Within the final 25 movements, pseudo-randomly distributed throughout them, six of the target dots would appear to ‘jump’ to the next target position before the participant had reached it; assessing participant’s capacity for making online corrections. ‘Baseline’ trials were denoted as the first 50 trials where no jump events had occurred. ‘Embedded’ trials were the normal trials in the last 25 movements with ‘jump’ trials being when the targets changed location mid-movement.

*Tracing*: this final subtest required participants to move their stylus along a static path between designated start and finish points, trying to trace as accurately as possible, whilst staying within the guidelines of the path (see Fig. 1c). The trial was initiated and the whole path appeared when the stylus was placed on the ‘start’ block for 1 s. A hollow box moved along the pattern to provide a guide for the ideal speed of movement. To try to avoid either very slow accurate tracing or rapid inaccurate tracing the participants were instructed to keep the stylus within the box throughout the trial (time for box to move from start to finish = 35 s). The trial was finished when the ‘finish’ block was reached. Participants completed three repetitions of two different tracing paths. The two paths were presented in alternate trials, totalling six tracing trials within the subtest.

### Postural measures

Postural movement was calculated using a custom built motion-capture rig and force platform, specifically designed to be used in schools. The rig comprises a stereo-camera motion-capture system that measures the 3D position of an infra-red (IR) diode at 60 Hz. A battery-powered IR diode was placed on a light, inflexible plastic brace placed on the child’s head, which provided a measure of head movement (HM). In addition to the measure of movement at the head, a Nintendo Wii Fit board was used to simultaneously monitor the participant’s centre of pressure (COP) at 60 Hz.

On another testing session (separated by at least 2 days from the manual control assessment) participants were asked (i) to stand with their feet shoulder width apart with their eyes closed for 30 s, then (ii) to stand with their feet shoulder width apart with their eyes fixed on a target placed 1 m away at eye level. During both conditions (hereafter referred to as ‘eyes closed’ and ‘eyes open’, respectively), the participants were constantly observed to ensure compliance. HM data were filtered using a 10 Hz dual-pass Butterworth filter, and the COP data were filtered using a wavelet filter described in Flatters et al. (2014a). After filtering, the 3D and 2D path lengths subtended by the IR diode and COP, respectively, were calculated (in mm) for each 30 s trial. Allowing time for measurement equipment set-up and rest-breaks, this session lasted approximately 3 min.

### Defining outcomes measures

*Postural measures*: we wished to analyse both head movement (HM) and centre of pressure (COP) variables separately and also to create a composite measure of these two variables, to provide an index of overall postural stability. Shapiro–Wilks tests indicated normality assumptions were met for HM and COP measures (*p*’s > 0.05). Thus, z-score transformations could be used to convert participants’ HM and COP scores to a unified scale. This then allowed for a mean of these two scores to be calculated, giving a ‘composite posture’ score. In order to control for the well-established age differences in motor control, we experimented with three different approaches to standardisation. First, each participant’s scores on HM and COP were standardised in relation to their means and standard deviations within the respective school years within the sample—(Years 1, 2, 3, 4, 5 and 6). Second, participants were grouped based on the year of birth and standardised in relation to each group’s means and SDs. For this, the following groups were used: 2009–2008; 2007–2006; 2005–2004; 2003–2002; and 2001–2000. Finally, scores were standardised relative to the entire sample and age included as a covariate in subsequent statistical analysis. Irrespective of the approach taken the same pattern of results was observed during analysis, demonstrating the robustness of the results. For conciseness, from here on, we only report results in which z-scores were calculated based on the first approach described (standardisation by school year). Note that age standardisation, when z-scoring, was also used with respect to all the following fine motor control measures.

*Manual control measures*: for every trial within every subtest of the C-KAT battery, the KAT software recorded the position of the stylus at a rate of 120 Hz and at the end of each testing session, these raw positional data were filtered using a 10 Hz dual-pass Butterworth filter. From these filtered time-series, a wide variety of spatial, temporal and frequency-based kinematic metrics could be calculated (see Culmer et al. 2009). To avoid data mining, only a specific a priori determined subset of all the potential variables was analysed. These were selected on the following criteria: (i) variables had to be normally distributed or responsive to transforms that enforced this (e.g. reciprocal, natural log). This legitimated z-score transformations of such variables, in turn allowing multiple outcomes relating to given subtests to be averaged to give composite scores for each subtest. (ii) Variables had to be at least moderately correlated with age (*r* > 0*.3)*, implying they were a meaningful measures of some characteristic of the development of fine motor control. (iii) The variables needed to relate to a measure that directly indicated performance on the task (as per explicit instructions to the children and implicit within the task design). Application of these criteria meant that the following kinematic variables were selected as outcomes for the respective C-KAT subtests.

For the *tracking* subtest, the spatio-temporal accuracy of the participant at each sampled time point was measured as the two-dimensional distance from the stylus to the dot centre (i.e. root-mean-square error [RMSE]). Across data points, a mean value for RMSE was calculated for each of the six experimental conditions (i.e. one per speed [slow, medium, fast] for both background conditions [without guideline, with guideline]). To capture the spatial accuracy of the shape subtended during pursuit, a second metric (path accuracy [PA]) was calculated, as the mean of the minimum distances from input to the ideal path across all data points (within each condition). These twelve measures of RMSE and PA (i.e. two metrics, three speeds and two background conditions) had reciprocal transforms applied to normalise the distributions and were then converted to standardised z-scores. A composite score for tracking was calculated as the arithmetic average of these twelve z-scores.

For the *aiming* subtest, median values for both the reciprocal movement time (MT) and the log normalised jerk (NJ) of the aiming movements made within each of the three experimental conditions were calculated separately (i.e. baseline, embedded and jump conditions). These six values were then z-score standardised and a composite score for the aiming subtest calculated by averaging these six standardised scores.

For *tracing*, the minimum 2D distance between the idealised reference path and the stylus was calculated for each sampled time point within a trial. For each of the six trials, the arithmetic mean of these values was taken as a measure of shape reproduction accuracy, termed path accuracy (PA). Despite continuous monitoring of the participants by the experimenter, a number of participants were unable to adhere to the instructions to stay within the moving on-screen box with their stylus, whilst tracing. Thus, interpretation of participants’ accuracy during these trails was potentially confounded by a lack of standardisation for their speed. Consequently, in order to control for variation in time, a ‘penalised path accuracy’ (PPA) metric was calculated that adjusted PA score with respect to movement time (MT). The ideal trial time, including the 1 s delay at the onset of the trial, was 36 s. To normalise path accuracy for task time, PA was inflated by the percentage that participants’ *actual* MT deviated from the ideal 36 s value. For each of the six trials, the PPA value a reciprocal transformation was applied to normalise its distribution before being z-score transformed and a composite performance score for this subtest calculated as the mean of these six values.

Finally, an *overall battery score* for the C-KAT was calculated as the arithmetic mean of the respective tracking, aiming and tracing composite scores.

## Results

We first explored the relationship between postural stability and manual control ability using the postural composite outcome measure and the C-KAT overall battery score, when these global scores were calculated from z-scores standardised relative to the entire sample (i.e. not adjusted for age). A strong relationship was found between these variables (*r* = 0.62 (278), *p* < 0.001). The strong correlation is consistent with previous findings that show large age effects for both postural measures (Flatters et al. 2014a; Flatters et al. in press) and performance on the manual control battery (Flatters et al. 2014b). The more interesting question was whether this relationship would still be observed after we controlled for age.

To begin exploration of the age-controlled relationship between posture and fine motor control we, for the remainder of our analyses, examined outcomes which were z-score standardised in relation to means and standard deviations within school years (i.e. age adjusted). Using these, performance on the HM, COP and the postural composite outcome measures were correlated separately against (i) the C-KAT overall battery score and (ii) each of the composite scores for the batteries’ subtests (i.e. tracking, aiming and tracing), using Pearson’s correlation coefficients (Table 1). Separate analyses of eyes-open and eyes-closed posture outcomes were conducted, as well as analyses that averaged performances across these two conditions. The strength of correlations for all the postural measures with all the C-KAT measures was similar across both posture conditions and therefore only the averages across these conditions (for HM, COP and postural composite) were analysed further. In the most reductive correlation (i.e. one overall score for posture vs. one overall score for fine motor control), the postural composite score showed a significant correlation with fine motor control (C-KAT) battery score (*r* = −0.27 (278), *p* < 0.001). In agreement with this, performance on each of the three C-KAT subtests showed small-to-moderately sized negative correlations (0.1 < *r* < 0.3, as classified in Field et al. 2012) with both HM and COP outcomes. However, associations were generally much weaker between posture and performance on the aiming subtest (*r* ~ −0.1). Also, HM but not COP was significantly associated with tracing performance. The fact that all correlations were negative indicates that as performance on the C-KAT battery outcomes improved (higher scores) postural instability fell (i.e. lower HM, COP and postural composite scores).

**Table 1:** Correlations between overall C-KAT battery scores and measures of postural stability, across eyes-open and eyes-closed conditions and a mean average of both

	C-KAT battery composite scores			
	Overall	Tracking	Aiming	Tracing
*Eyes*-*closed posture condition*				
Head movement	−0.28***	−0.20***	−0.13*	−0.32***
Centre of pressure	−0.14*	−0.15*	−0.11	−0.08
Postural composite	−0.24***	−0.20***	−0.14*	−0.23***
*Eyes*-*open posture condition*				
Head movement	−0.26***	−0.23***	−0.10	−0.28***
Centre of pressure	−0.17**	−0.23***	−0.08	−0.11
Postural composite	−0.24***	−0.26***	−0.10	−0.22***
*Mean for both posture conditions*				
Head movement	−0.29***	−0.23***	−0.12*	−0.32***
Centre of pressure	−0.18**	−0.22***	−0.11	−0.11
Postural composite	−0.27***	−0.26***	−0.13*	−0.25***

*ns* not significant (*p* > 0.05)

* *p* < 0.05; ** *p* < 0.01; *** *p* < 0.001

**Fig. 2:**
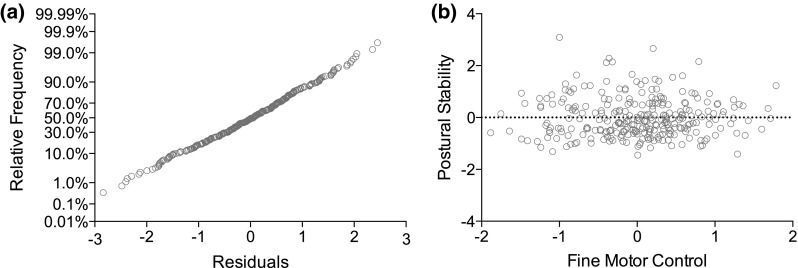
Model residuals: **a** cumulative distributions of the standardised residuals in the model plotted on the probability axis indicate normality; **b** residuals plotted against fitted values for the simple linear regression model

**Fig. 3:**
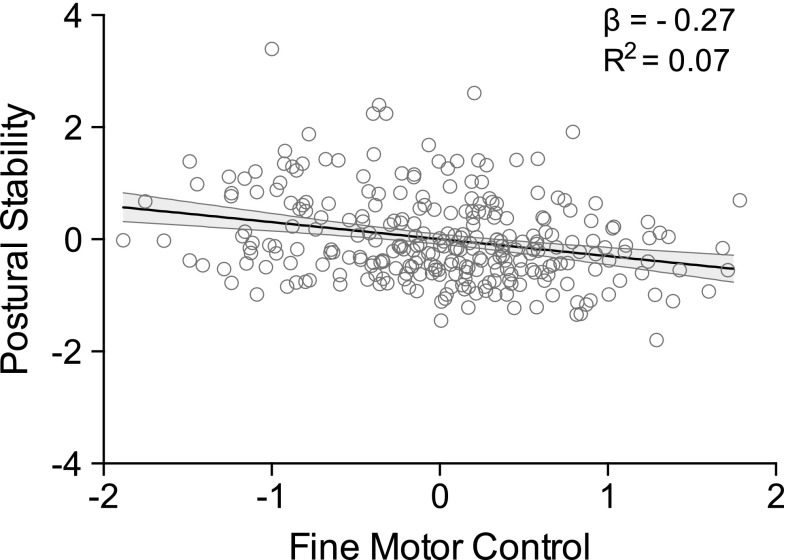
Simple linear regression analysis indicates that gross motor aptitude could significantly predict fine motor control performance (*b* = −0.24, *β* = −0.27, *t*(277) = 4.62, *p* < 0.001), with the predictor variable able to explain 7 % of the total variation in fine motor control performance (*R* ^2^ = 0.07, *F*(1, 276) = 21.37, *p* < 0.001). *Shaded area* represents 95 % confidence interval of the regression line. Abscissa shows standardised fine motor control performance and ordinate represents standardised scores on the composite measure of postural stability

Informed by this correlational analysis, an initial simple linear regression analysis was conducted to explore whether overall postural stability scores significantly predicted overall manual control performance. The postural composite score was used as the predictor variable, and the overall C-KAT battery score was used as the outcome variable for the simple linear regression model. A scatter plot of the data indicated that the assumption of linearity was reasonable, whilst the cumulative distributions plot of the standardised residuals in Fig. 2a supported the assumption of normality. Plotting the residuals against the fitted values (Fig. 2b) suggested no violation of the assumption of constant variance of the random errors. Results of the simple linear regression model (Table 2) indicate that fine motor control could be predicted from children’s postural control abilities (*b* = −0.24, *β* = −0.27, *t*(277) = 4.62, *p* < 0.001). Specifically, the composite measure of postural stability explained a modest but significant proportion of variance in fine motor manual control, as indexed by the overall C-KAT battery score (*R* ^2^ = 0.07, *F*(1, 278) = 21.37, *p* < 0.001). See Fig. 3 for the simple linear regression plot.

**Table 2:** Simple linear regression model for overall C-KAT battery score

Explanatory variable	*b*	Standard error	*β*	*t*	*p*
Constant	0.01	0.04		0.28	0.78
Postural composite	−0.24	0.05	−0.27	−4.62	<0.001

To provide a more detailed analysis of the separate underlying relationships between the manual and postural measures included in the preliminary ‘composite’ analysis, forced-entry multiple linear regression modelling was used to assess the extent to which HM and COP each independently predicted fine motor manual control performance; on each of the C-KAT subtests and the overall battery as a whole. Four multiple linear regression analyses were computed using HM and COP as independent variables and each analysis specifying one of the four following outcomes as its dependent variable: overall C-KAT battery score, tracking composite, aiming composite or tracing composite. All models satisfied assumptions of independence and multicollinearity. The results of these four models are reported in Table 3 and show that HM but not COP significantly predicted tracking and tracing performance, as well as overall battery score (although it should be noted that COP’s contribution to explaining variation in tracking performance did approach significance, *p* = 0.051). Meanwhile, neither postural measure explained a significant amount of the variance for the aiming subtest. Nor was the explanatory power for any of the models particularly high (*R* ^2^ ranging from 0.01 to 0.10), judged against conventional benchmarks for quantifying effect size of *R* ^2^ (i.e. ‘small’ effect size = 1 % of the variance explained; ‘medium’ effect size = 9 % of the variance—as classified in Field et al. 2012).

**Table 3:** Multiple linear regression models of C-KAT battery performance (overall and on individual subtests) predicted by head movement and centre of pressure

Dependent variable	Explanatory variables	*R* ^2^	*b*	Standard error	*β*	*t*	*p*
C-KAT overall		0.08					
	Constant		0.01	0.04		0.28	0.778
	Head movement		−0.20	0.05	−0.27	−3.97	<0.001
	Centre of pressure		−0.03	0.06	−0.03	−0.47	0.636
Tracking composite		0.06					
	Constant		<0.01	0.05		−0.05	0.957
	Head movement		−0.13	0.06	−0.16	−2.29	0.023
	Centre of pressure		−0.12	0.06	−0.14	−1.96	0.051
Aiming composite		0.01					
	Constant		<0.01	0.05		−0.07	0.944
	Head movement		−0.08	0.06	−0.09	−1.29	0.197
	Centre of pressure		−0.06	0.07	−0.06	−0.81	0.418
Tracing composite		0.10					
	Constant		0.04	0.06		0.71	0.478
	Head movement		−0.39	0.07	−0.37	−5.49	<0.001
	Centre of pressure		0.10	0.08	0.09	1.29	0.197

## Discussion

It is well known that head, hand and posture are functionally related. For example, recent empirical data have shown that postural stability is found to vary as a function of task demands (Haddad et al. 2010; Flatters et al. 2014c). However, this raises of the question of whether the development of these control systems is largely separate or highly determinant. As outlined in the introduction, there are three broad positions with respect to the relationship between these skills that are mutually exclusive: (1) postural control and fine motor skills are completely independent developing processes requiring absolute taxonomic separation when examining performance; (2) postural control and fine motor skills are highly correlated attributes that reflect an underlying ability; and (3) postural control and fine motor skills are separate processes that nonetheless affect each other’s development through their co-dependent functional combination across various tasks.

Previous research seeking to examine the developmental relationship between postural stability and manual skill has produced unclear results (Loria 1980; Case-Smith et al. 1989; Rosenblum and Josman 2003; Wang et al. 2011). This is perhaps unsurprising when one considers that existing studies have often collected data from relatively small populations and relied on subjective measures. To address these issues, the present study used objective measures of postural stability and manual dexterity and collected data from a reasonably large population of school children. In initial analysis of composite measures, results showed a strong relationship between postural stability and fine motor manual control before correcting for age. Following correction for age, these relationships remained significant, but were relatively small in magnitude. Overall, a composite postural stability measure predicted 7 % of the variance in a composite measure of fine motor control, when age was controlled through the use of standardised scores. Further exploration of the data indicated that certain postural measures (head movement but not centre of pressure) significantly predicted modest amounts of variation in performance on specific subtests of the manual control battery (tracking and tracing). The greater sensitivity of the head movement measure can be explained by noise in the respective signals. Centre of pressure acts as a proxy for changes in centre of gravity but these changes do not map directly to postural maintenance (Flatters et al. 2014b). It can be argued that the primary goal of the human postural system is to provide a stable platform for the acquisition of visual information—i.e. holding the head steady. Thus, it is perhaps not surprising that head movement provides the most useful measure of postural stability when maintaining posture with the eyes closed (Flatters et al. 2014b).

The findings from the present study allow us to reject the hypothesis that postural and manual control abilities are completely independent (Position I). Importantly, the postural and fine motor measures were taken at different time points (separated by at least 2 days), an arrangement that provides a strong test of the hypothesis that the different skill measures will have a correlational relationship. Nevertheless, the majority of the variance in fine motor control was not explained by the ability to maintain posture (only between 1 and 10 % depending on the regression model). The ability of the postural measure to predict variation in manual control also showed a degree of task-specificity (see Table 3). For example, posture measures did not predict performance on the manual aiming subtest. This supports rejection of the hypothesis that a single attribute (a postulated ‘motor ability’ construct) unilaterally underpins ‘gross’ and ‘fine’ motor control (Position II).

It may be that testing younger children than those included in our sample would yield stronger relationships between the posture and manual variables, with these skills only diverging in their developmental trajectories over time (e.g. Horn et al. 2006). This concurs with the strongest evidence for such a relationship in the earlier literature having been found in research conducted in infants (Wang et al. 2011). Nonetheless, the present study shows that the relationship between postural control and manual ability is modest above the age of 3 years. This may be because manual skill can develop despite poor postural control if external objects are used to help stabilise posture. For example, once a child is able to sit on a chair they can use the stability of the chair to reduce the postural control demands (e.g. children learn to write whilst seated at a desk).

The picture that emerges from the present study is one where the development of postural control and the development of manual control have a degree of task-specific co-dependency (Position III). The interactions between reaching for an object and postural maintenance have been described previously in the context of dynamic systems theory where development is characterised by evolving and dissolving patterns of dynamic stability rather than a set of linear progressions towards mature behaviour (Thelen and Spencer 1998; Fallang et al. 2005; Haddad et al. 2013). In this formulation, postural and fine motor control mechanisms can be viewed as independent dynamical processes, which often interact in the course of development. These interactions are marked by the emergence of more complex ‘higher level’ coordinated motor actions. This conception is consistent with longitudinal studies of development and appears to capture the findings of the present study in an elegant manner.

Some limitations of the current study are worth noting. When generalising from these results, the likelihood that relationships are moderated by a degree of task-specificity, needs to be kept in mind. The three manual tasks we used evaluated manual performance on a variety of uni-manual fine motor tasks which required in-hand manipulation of a stylus, whilst the postural assessments measured an important aspect of overall gross motor control: static balance. Consequently, these assessments can be taxonomically classified, respectively, as measures of *fine* and *gross* motor control. However, this does not mean that either assessment should be viewed as a definitive evaluation of a participant’s fine or gross motor control skills in their entirety. The tasks we used assessed ability to respond to certain fundamental challenges that commonly arise when performing motoric tasks (e.g. visual-guided tracking and aiming, manipulating an object with ones hands, maintaining a stable platform). Thus, the relationships we observe are likely to be indicative of the general pattern of association between posture and manual control. However, when extrapolating from these findings to hypothesise the degree of relationship likely to exist between a novel pair of fine and gross motor tasks, it is important to note that the relevance of this studies’ findings to said novel set of tasks will be influenced by the degree to which these novel tasks tap into the same functional challenges as the tasks reported here. Similarly, it is worth noting that postural control during standing and manual control, whilst seated pose different postural demands. This study was concerned with the *underlying* abilities of children with regard to their postural and manual control abilities, one avenue for future research could be to explore the relationship between these skills in tasks that pose equivalent postural demands.

The findings of this study have practical implications, specifically relating to the assessment of motor ability in children. A number of standardised movement assessment batteries for children (e.g. the MABC-2) test manual dexterity separately to postural control. This arrangement has lacked empirical justification in the past, but our results provide a clear rationale for this division, corroborating recent confirmatory factor analysis demonstrating the construct validity of the MABC-II (Schulz et al. 2011). Our findings also question the usefulness of combining scores from tests of manual dexterity and postural control. A number of assessment batteries provide a composite score that indicates a child’s overall motor proficiency. The implicit assumption in such practice is that there is an underlying construct of ‘general motor ability’. The results of the present study suggest that this construct may not have validity. Indeed, children who experience difficulties in motor development often have a deficit in fine, but not gross motor skills or vice versa (Visser 2003; Zwicker et al. 2012). On this basis, it can be argued that the production of a combined motor performance score is not useful and might actually mask a profound deficit in one domain. We would argue that it is more useful to provide these scores separately and flag when a child is performing below an acceptable level on either (e.g. the fifth percentile for chronological age; Blank et al. 2012) and intervene accordingly.

The argument for presenting postural measures separately from manual skill scores when assessing children does not imply that those children with the most profound movement problems will not have difficulties in both domains. It may be that children with pathological difficulties such as cerebral palsy and development coordination disorder (DCD) struggle with both gross and fine motor tasks. It is easy to imagine that deficits in these different systems interact to create considerable difficulties (the ‘double whammy’) when engaging in activities of daily living (ADLs). It is also possible that a deficit in either domain might act as a barrier to a particular ‘higher-order’ activity (e.g. pulling on a sock when standing might be made difficult by poor balance or poor dexterity).

A better understanding of the relationship between deficits in posture, manual dexterity and ADLs would allow more tailored interventions for children with movement problems. Our findings suggest that poor performance in one domain is not necessarily a reliable indicator of difficulties in another domain. This suggests that a child with manual dexterity problems may not benefit from a therapeutic approach that encourages improved posture (if the child has no postural difficulties). It follows that children should be assessed in depth to produce a profile of their strengths and weaknesses. This would allow targeted therapy so the child with postural difficulties could receive help with maintaining balance, whereas the child with manual control problems could obtain help directed towards improving their manual dexterity. We note that the objective measures described in this paper would allow therapists to provide such targeted interventions.
